# Determinants of availability of tracer essential commodities in Kitui County Referral Hospital, Kenya: a cross-sectional mixed-methods study

**DOI:** 10.11604/pamj.2025.52.81.48680

**Published:** 2025-10-22

**Authors:** Jackson Munyaka Kalola, Atei Kerochi, Alice Theuri

**Affiliations:** 1 Department of Health Systems Management, Mount Kenya University, Kabati, Kenya,; 2 Department of Public Health, Mount Kenya University, Garissa, Kenya,; 3 Department of Food Science, Nutrition and Technology, South Eastern Kenya University, Kitui, Kenya

**Keywords:** Essential, drugs, shortages, inventory control, cross-sectional studies, health services accessibility

## Abstract

**Introduction:**

uninterrupted availability of tracer essential health commodities is critical for service readiness and resilient healthcare delivery. Despite reforms, Kenyan public hospitals, including Kitui County Referral Hospital continue to experience recurrent stock-outs of essential items.

**Methods:**

this cross-sectional mixed-methods study assessed the availability of 155 tracer commodities across 22 departments at Kitui County Referral Hospital from July 2023 to June 2024. Quantitative data were collected using structured stock audits guided by the Ministry of Health 647 checklist, and qualitative insights from 12 key informant interviews. Data were analyzed using SPSS v26 and thematic analysis. Triangulation enhanced data analysis and interpretation.

**Results:**

only 2 of 22 departments (9.1%) met the WHO-recommended 80% availability threshold. Availability ranged from 29% in the non-pharmaceutical store to 100% in the dental and nutrition departments. Critical shortages affected high-demand areas like the intensive care unit (73%) and maternity unit (67%), with frequent stock-outs of intravenous solusets and pediatric electrodes. Supply bottlenecks included delayed deliveries from the Kenya Medical Supplies Authority (51-60% order fulfillment) and reliance on manual inventory systems. Only 22.7% of staff had inventory management training, but trained departments showed significantly higher availability (p = 0.029). Supervisory oversight and forecasting tool use also influenced availability.

**Conclusion:**

tracer essential commodity availability at Kitui County Referral Hospital remains inadequate due to systemic supply chain inefficiencies and weak inventory management. Prioritizing staff training, digital inventory systems, and stronger supervision are actionable strategies to reduce stock-outs and support Kenya´s Universal Health Coverage goals.

## Introduction

Uninterrupted availability of essential medicines remains a critical indicator of health system strength and service readiness [1,2]. Essential medicines, as the World Health Organization (WHO) conceptualizes them, are those that satisfy the priority health care needs of the population. Their continuous availability across all levels of care is a prerequisite to the provision of equitable, timely, and effective services [3]. However, in most low- and middle-income countries, such as Kenya, public health facilities consistently run out of essential health commodities [4,5]. Such stock-outs contribute to poor quality of care, treatment delays, and erosion of public confidence in health systems [6].

To combat the chronic issue of shortages of essential medicines, the Ministry of Health in Kenya adopted the Kenya Essential Medicines List (KEML), as per guidelines outlined by the WHO [7]. The list designates a subset of tracer commodities, including essential medicines, supplies, and equipment, which are key determinants of a functional health supply chain and determine the readiness of facilities to provide care [8]. These commodities form the core of standard patient care: items used often, essential for diagnosis and treatment, and indicative of a hospital's ability to coordinate its logistics and inventories [9]. Their regular presence is a sign of a responsive and coordinated system, and repeated stock-outs tend to reveal structural or operational issues [3,10].

At the center of Kitui County's health system is Kitui County Referral Hospital (KCRH), the primary public referral hospital, caring for a sizable and increasing patient load. In spite of its key position, KCRH has grappled with recurrent stock-outs of these key tracer commodities [3,5]. The root causes of these long-standing shortages, nonetheless, are still poorly understood. As per both frontline personnel and county health reports, the underlying factors are numerous: ineffective procurement procedures, restricted staff training in inventory management, poor utilization of real-time data for projecting needs, budgetary limitations, and inconsistent supervisory oversight [4,9]. These issues at KCRH reflect overarching systemic problems observed throughout Kenya's devolved public health system [7,8].

Existing literature predominantly examines national or county-level medicine availability, with limited focus on facility-specific contextual dynamics [1,10]. Such a knowledge gap does not consider the critical contributions of institutional structures, human resources, and day-to-day service delivery pressures that have a direct bearing on commodity availability in high-volume referral hospitals such as KCRH [3,4]. Knowledge of these localized determinants is critical for the development of targeted, evidence-based interventions [3].

This research explores the determinants of the availability of tracer essential commodities at KCRH, a high-volume public referral hospital in Kenya. Through an in-depth look at one high-volume hospital, the study aims to produce context-specific evidence to guide policy formulation, enhance facility-level inventory management, and increase supply chain stability in comparable resource-poor settings.

Objectives: the study aimed to: (1) evaluate the current availability of tracer essential commodities at KCRH; (2) examine the human resource factors associated with commodity availability; and (3) evaluate the organizational determinants affecting the consistent availability of tracer essential commodities in the hospital.

## Methods

**Study design:** this study employed a cross-sectional mixed-methods design to investigate the determinants of tracer essential commodity availability at KCRH. Through the combination of quantitative and qualitative methods, the study gained a holistic picture of supply chain performance at the facility level [10,11].

**Study setting and population:** the study was conducted at KCRH, the primary public referral facility in Kitui County, Kenya. A county is a politically and economically semi-autonomous region in Kenya. As a referral hospital, KCRH serves a catchment population of over 1.1 million people and handles referrals from all areas of the region. The facility includes multiple departments: pharmacy, inpatient wards, outpatient units, radiology, laboratory, intensive care unit (ICU), nutrition, and central stores, and manages an average of over 20,000 patient visits monthly, reflecting consistently high patient volumes [4]. The target population for this study consisted of tracer essential health commodities tracked across key hospital departments, as defined by the Ministry of Health´s MOH 647 reporting tool [12]. The units of analysis were the hospital departments directly responsible for managing, requisitioning, dispensing, or utilizing these commodities, including pharmacy, inpatient and outpatient services, ICU, laboratory, nutrition, and central stores.

**Sample size and sampling technique:** for the quantitative component, a sample size estimation formula and adjusting for the finite population using the finite population correction (FPC) formula resulted in a sample size of 155. The health facility was stratified, where each department formed a stratum. Sample size allocation for each stratum was proportionate to its size. A simple random sampling technique was applied to select participating tracer items in each stratum. The 155 tracer commodities represent 59.4% of the total 261 tracer items across 22 departments, as per the MOH 647 tracer commodity list.

For the qualitative component, 12 key informants were purposively selected based on their operational roles in supply chain management, inventory control, and decision-making within the facility. These participants included departmental heads and staff directly involved in managing tracer essential commodities. The selection of key informants was guided by role-based relevance, which ensured comprehensive coverage of the organizational and human resource perspectives related to tracer commodity availability across departments [13].

**Data collection tools and variables:** the dependent variable was the availability of tracer essential commodities, which looked at the availability of tracer essential commodities. The independent variables were human resources factors, which included staff turnover rates, qualifications, and training. Organizational factors included: forecasting commodity needs, procurement processes and practices, budgeting, and inventory management.

Quantitative data collected using a structured abstraction tool and observation checklist, whose development was informed by the MOH 647 tool [12] and the Kenya Essential Medicines List (KEML) [8]. Qualitative data were collected using an interview guide to explore procurement processes and practices, inventory control practices, supply chain governance, and funding mechanisms.

**Data analysis:** quantitative data were analyzed using IBM SPSS version 26. Descriptive statistics, including frequencies and percentages, were used to summarize the availability of tracer essential commodities and relevant categorical variables. Chi-square tests were used to assess associations between independent variables (e.g., staff training, turnover, patient volumes) and commodity availability. A p-value of <0.05 was considered statistically significant [14,15]. Qualitative data were transcribed verbatim and analyzed using manual thematic analysis. A piloted structured interview guide was used to ensure consistency during data collection. Transcripts were reviewed repeatedly for familiarization, and inductive coding was conducted based on recurring patterns in participant responses [13]. Codes were grouped into themes that aligned with the study objectives, including organizational structures, human resource capacity, logistical bottlenecks, and demand-related challenges. Thematic analysis was supported using NVivo software [13]. Findings from the qualitative data provided contextual insights that complemented the quantitative results and enhanced understanding of the determinants influencing tracer essential commodity availability [4,10]. Triangulation of quantitative and qualitative findings strengthened the study´s internal validity by integrating measurable trends with stakeholder perspectives [10,11].

**Ethical considerations:** ethical approval was obtained from the Mount Kenya University Ethics Review Committee (Ref No. MKU/ERC/1601), with a research permit issued by the National Commission for Science, Technology and Innovation (NACOSTI) (Ref No. NACOSTI/P/22/13713). Additional administrative permissions were secured from the Kitui County Director of Health and the Medical Superintendent of KCRH. Written informed consent was obtained from all participants, and permission was granted for audio-recording of interviews. Confidentiality and anonymity were maintained throughout the study, with no personal identifiers recorded or disclosed during data analysis or reporting.

## Results

**Figure 1:**
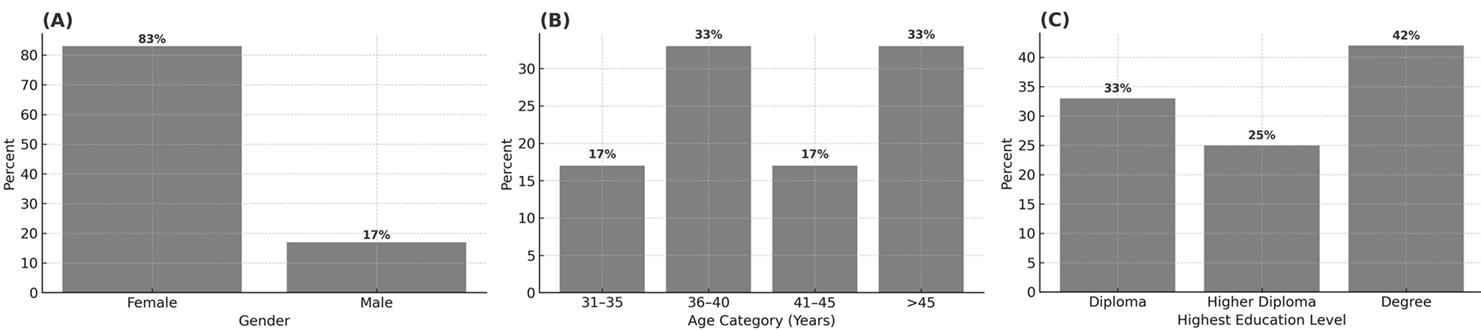
demographic characteristics of study participants at Kitui County Referral Hospital, Kenya (February 2025)

**Demographic characteristics of the participants:** participants were drawn from 22 study units, including ward in-charges, procurement, and supply chain staff. Most were female, aged between 36 and 45 years, with a mean age of 43. Educational qualifications ranged from a diploma to degree level, and the average work experience was 14 years. All were health professionals involved in essential commodity management. Notably, 12 participants were ward in-charge nurses, providing key insights on departmental stock availability. A summary of the participant demographics is presented in Figure 1.

**Availability of tracer essential commodities:** at the time of assessment, only two (9.1%) out of twenty-two departments at KCRH achieved the WHO-recommended threshold of ≥80% availability of tracer essential commodities. Overall availability levels varied significantly across departments. The dental and nutrition departments recorded 100% availability, while high-acuity areas such as the ICU (73%), maternity ward (67%), and surgical ward (64%) demonstrated moderate availability of critical items. The drug store reported 48% availability, while the non-pharmaceutical store recorded the lowest availability at 29%. These findings indicate that 90.9% of departments (20 out of 22) did not meet the WHO benchmark, suggesting a facility-wide need to improve stock management and supply chain efficiency. Full departmental availability results are presented in Table 1.

**Table 1:** tracer commodity availability by hospital department at Kitui County Referral Hospital, Kenya (July 2023-June 2024)

Department	Items assessed (n)	Items available (n)	Availability (%)	Common items are out of stock
Drug store	40	19	48	Anti-D, salbutamol, methylated spirit
Non-pharm store	28	8	29	Zinc oxide, sutures, and urine bags
Laboratory department	9	6	67	Formaldehyde, urinalysis strips
Radiology unit	3	2	67	CT scan films
Dental unit	2	2	100	None
Renal unit	2	1	50	None
Nutrition unit	2	2	100	None
Inpatient departments (wards and theatre)	23	14	61	Ambu bags, blood giving sets
Outpatient departments (OPD, MCH, Amenity)	23	12	55	Pediatric antibiotics (e.g., amoxicillin syrup)
ICU	15	11	73	Pediatric electrodes, syringes
NBU	8	5	63	Ambu bags, intraosseous needles, oxygen masks/nasal prongs

This table presents the number and percentage of tracer essential commodities available across departments within a 12-month period. It details the most frequently out-of-stock items to identify departments experiencing persistent shortages. The aim is to evaluate department-level patterns in tracer commodity availability to inform targeted supply interventions. Availability (%) calculated as (items available / items assessed) x 100. OPD: outpatient department; MCH: maternal and child health; ICU: intensive care unit; NBU: new born unit

**Human resource factors influencing availability:** departments led by staff who had received specialized training in inventory or commodity management demonstrated significantly higher tracer commodity availability (p = 0.029). Although only 22.7% of departmental in-charges had such targeted training, these departments consistently maintained better stock levels compared to those led by untrained personnel. Other human resource factors significantly associated with improved availability included routine supervisory visits (p = 0.026), longer staff experience exceeding five years (p = 0.024), and low staff turnover (p = 0.026). Additionally, departments that employed consumption-based forecasting methods showed significantly better commodity availability (p = 0.021) (Table 2).

**Table 2:** association between human resource factors and availability of essential commodities at Kitui County Referral Hospital, Kenya (July 2023-June 2024)

Human resource factors	Category	Adequate availability (≥80%)	Inadequate availability (<80%)	Total n (%)	χ^2^	p-value
Staff training in inventory	Trained	4	1	5 (22.7%)	4.76	0.029*
	Not trained	2	15	17 (77.3%)		
Years of experience	>5 years	5	8	13 (59.1%)	5.10	0.024*
	≤5 years	1	8	9 (40.9%)		
Staff turnover	Low (≤10%)	6	7	13 (59.1%)	4.98	0.026*
	High (>10%)	0	9	9 (40.9%)		
Gender	Male	2	0	2 (9.1%)	1.63	0.201
	Female	4	16	20 (90.9%)		
Age of the in-charge	≥35 years	4	10	14 (63.6%)	0.88	0.348
	<35 years	2	6	8 (36.4%)	2.12	0.232

This table analyzes associations between human resource factors and the availability of essential commodities. The analysis employs chi-square statistics to identify significant relationships, offering insights into staff-related determinants of commodity stock adequacy. *p < 0.05; statistically significant. Total n (%): number and percentage of the 22 departments included in the analysis.

Qualitative interviews provided further context to these findings. Participants frequently cited frequent staff rotations without adequate handovers, inconsistent supervision, and poor interdepartmental coordination as critical contributors to weak inventory control. The absence of structured leadership oversight in enforcing inventory management protocols further compounded the human resource challenges, highlighting gaps in both technical capacity and accountability structures. These facility-level issues undermined the ability of staff to ensure timely ordering, tracking, and redistribution of tracer essential commodities.

**Organizational factors affecting availability:** procurement and delivery inefficiencies emerged as significant organizational-level barriers to the consistent availability of tracer essential commodities at KCRH. Quantitative data revealed that the Kenya Medical Supplies Authority (KEMSA), the facility´s primary supplier, fulfilled only 51-60% of the hospital´s requested orders during the assessment period. Departments were having 2-3-week lead times, especially during weekends, holidays, and quarter-end. Very often, products that were consistently behind schedule were ambu bags, syringes, and blood giving sets.

Qualitative interviews supported these findings, highlighting inadequate procurement budgets, fragmented procurement processes that involved multiple suppliers, and limited coordination between the procurement office and clinical departments. Several departments reported that despite central warehousing of commodities, internal delays during distribution resulted in spot shortages within points-of-care. In an effort to mitigate these delays, employees were seen to exchange interdepartmentally or even have patients buy necessary items outside during extreme circumstances.

Additionally, continued hospital use of manual tracking methods to tally stock contributed to delays in reordering and accurate forecasting. Inability to sustain electronic stock instrument maintenance, inadequate supervision, and uneven execution of stock-control measures were cited by respondents as important factors eroding distribution and procurement effectiveness. Despite being influenced by external supplier limitations, most of the organizational weaknesses underscore KCRH´s internal process gaps. The inability to work effectively to procure, to demand timely redistributions, and to improve inventory configurations contributed significantly to commodity stock-outs.

**Service demand factors:** service demand patterns also influenced the availability of tracer essential commodities across departments. Qualitative data from key informants indicated that fluctuating patient volumes, particularly in high-acuity departments such as the ICU, maternity ward, and outpatient services, often led to sudden spikes in demand that were not adequately forecasted in procurement cycles. Staff reported that referrals from lower-level facilities, combined with seasonal disease outbreaks, placed additional pressure on already limited inventories, particularly for commonly used items such as IV fluids, antibiotics, and emergency kits. Departments with higher daily patient loads struggled to maintain optimal stock levels, particularly when combined with delayed restocking and a lack of buffer inventory.

These demand-side pressures were exacerbated by inadequate forecasting tools and methods, leading to frequent mismatches between available stock and real-time departmental needs. The lack of real-time data visibility further limited the hospital´s ability to respond promptly to demand surges. These findings highlight the need for more robust demand forecasting systems, better coordination between referring facilities and KCRH, and data-driven procurement planning to match service utilization trends with supply availability.

## Discussion

This study found that the availability of tracer essential commodities at KCRH was suboptimal, with wide variability across departments. Only two of the 22 departments met the WHO-recommended threshold of 80% availability, while others, including high-acuity areas such as ICU and maternity, fell significantly short.

The variation within KCRH, ranging from 29% in the non-pharmaceutical store to 100% in nutrition and dental departments, points to operational inefficiencies that are often concealed in aggregated national-level data. These results are consistent with findings by Ayako *et al*. who reported 63% average availability in select Kenyan counties [5], and Demessie *et al*. who found similar patterns of 50-70% availability in Ethiopia [11]. A Ministry of Health national survey found a 68% mean availability, indicating that the issue of tracer commodity availability is widespread and systemic even in facilities with better resource allocations [4].

Several human resource-related variables were associated with commodity availability. Availability was consistently higher in departments with better-trained personnel and routine oversight, reinforcing the importance of human resource investment. Qualitative interviews reinforced these results, identifying issues such as frequent staff rotations without proper handovers, poor interdepartmental coordination, and inconsistent supervision as key challenges. These results corroborate prior studies that emphasize training and supervision as critical enablers of inventory performance [8,10]. Insights from qualitative interviews contrast with some prior literature that emphasizes national-level supply chain constraints as primary drivers of shortages [2,5], suggesting a more substantial role for facility-level human capacity and oversight.

Beyond personnel, organizational inefficiencies also emerged as critical determinants of commodity availability. Departments utilizing consumption-based forecasting showed significantly improved availability (p = 0.021). Interview data revealed additional organizational challenges: fragmented procurement workflows, lack of structured supervision, manual inventory tracking systems, and delays in internal redistribution. The study found that only 51-60% of ordered items from the Kenya Medical Supplies Authority (KEMSA) were fulfilled, confirming earlier findings on supplier constraints as a contributor to stock-outs [1,4]. However, internal hospital factors appeared to exert an even greater influence. Such gaps were also observed in Muranga and Machakos counties, where weak departmental coordination was linked to poor inventory performance [6]. These findings support the literature that advocates for digital inventory systems and forecasting models to reduce shortages [9,16]. Compared to better-performing counties like Nairobi and Uasin Gishu [5], KCRH lagged due to limited use of digital tools and inconsistent leadership oversight.

Qualitative responses suggested that departments experiencing higher patient volumes, particularly OPD, maternity, and ICU, faced greater difficulty maintaining adequate stock. Unpredictable referral patterns, emergency admissions, and the absence of dynamic procurement adjustments were cited as contributing to recurrent shortages. These findings align with Demessie *et al*. who found that patient surges during epidemics or peak demand seasons often led to short-term commodity unavailability [11].

A key strength of this study is its mixed-methods design, which allowed triangulation of findings across stock audits, structured checklists, and interviews, enhancing data validity [12]. Use of standardized national tools such as the MoH 647 improved methodological consistency [11], while department-level focus enabled granular insights often missed in broader national studies [4]. However, limitations include the cross-sectional design, which restricts causal inference and omits seasonal variations in supply and demand [17]. Additionally, reliance on self-reported data introduced recall and desirability bias, though this was partially mitigated through cross-checking with physical stock records. Moreover, the lack of a digital inventory system constrained real-time data analysis and trend identification.

While prior studies often highlight supply delays from KEMSA as the main driver of stock-outs [1,2,5]. This study identified internal hospital operations, particularly human resource gaps, poor supervision, manual tracking, and coordination lapses, as equally influential. While referral facilities are typically presumed to perform better due to enhanced resources [3], this assumption was not supported at KCRH, where only two departments met the WHO availability benchmark. This finding emphasizes the need for facility-level diagnostics, which may uncover hidden inefficiencies masked in aggregated data [4].

Only two out of twenty-two departments at KCRH achieved the WHO-recommended threshold of 80% availability of tracer essential commodities. This finding underscores a critical gap in service readiness and reflects poor quality of care in the majority of hospital units. Improving the availability of these commodities is essential for enhancing the quality and reliability of healthcare delivery. The study demonstrates that enhancing availability involves more than timely deliveries from national suppliers like KEMSA [5]. Internal facility-level interventions, such as staff training in inventory management and routine supervisory monitoring, emerged as feasible and affordable levers for improving supply chain performance. These interventions are particularly relevant in resource-constrained settings, where large-scale system overhauls may not be immediately practical. The findings highlight simple, evidence-based measures that are critical to addressing persistent stock-outs, including digital inventory systems, formalized supervision, and targeted human resource development [16]. Even without national-level reforms, KCRH can achieve consistent access to tracer commodities by addressing these internal operational issues. This is in line with Kenya´s UHC goals because consistent access to essential medicines remains a core UHC indicator [7]. Systematic investment in facility-level capacity offers a practical and scalable path toward improving commodity availability and achieving equitable, quality healthcare delivery [4].

While this study offers critical insights at the facility level, important questions remain. Future research should explore tracer commodity availability across different facility tiers (levels 2 to 6) and geographical regions in Kenya to identify systemic versus localized challenges. Longitudinal studies evaluating the sustained effects of interventions such as digital inventory systems, structured supervision, and public-private supply partnerships could provide insights into scalable solutions [9,16]. Additionally, comparative analyses involving both public and private healthcare facilities may uncover further operational disparities affecting supply chain resilience [6].

## Conclusion

This study confirms that stock-outs of tracer essential commodities remain a chronic issue even in high-tier public hospitals like KCRH. Only two departments achieved the WHO-recommended 80% availability threshold, with critical departments such as maternity, surgical, and intensive care units consistently falling below this standard. Although extrinsic factors like delayed deliveries from central suppliers contributed to the stockouts, inherent facility-level frailties, namely poor staff training, absence of digital inventory systems, limited supervisory oversight, and lax interdepartmental coordination, surfaced as pivotal contributors. These operational shortcomings necessitate focused, facility-led solutions. Building staff capacity in inventory management, implementing digital stock control systems, and formalizing regular supervisory monitoring can provide pragmatic, affordable solutions to enhance commodity availability. These solutions are feasible within current resource limitations and constitute a vital avenue toward progressing Kenya's Universal Health Coverage ambitions by promoting uninterrupted access to life-saving medicines at the point of care.

### What is known about this topic

- Stock-outs of essential commodities remain a persistent challenge in Kenya´s public health facilities;
- Supply chain inefficiencies and delayed deliveries from national suppliers such as KEMSA frequently disrupt medicine availability;
- Staff shortages and limited health facility funding are commonly cited barriers to consistent commodity supply.

### What this study adds

- Staff training in inventory management and routine supervisory visits are significant predictors of tracer commodity availability;
- Internal facility-level factors, including leadership support, supervision, and interdepartmental coordination, substantially influence supply chain performance;
- Strengthening internal systems can produce measurable improvements in availability, even in the presence of external supply and funding constraints.
